# Development and Validation of a Risk Score for Predicting Post-acute Myocardial Infarction Infection in Patients Undergoing Percutaneous Coronary Intervention: Study Protocol for an Observational Study

**DOI:** 10.3389/fcvm.2021.675142

**Published:** 2021-05-28

**Authors:** Yuanhui Liu, Litao Wang, Yaowang Lin, Wei Chen, Zhengrong Xu, Pengyuan Chen, Yining Dai, Lihuan Zeng, Hualin Fan, Ling Xue, Jiyan Chen, Ning Tan, Chongyang Duan, Pengcheng He

**Affiliations:** ^1^Guangdong Provincial Key Laboratory of Coronary Heart Disease Prevention, Department of Cardiology, Guangdong Cardiovascular Institute, Guangdong Provincial People's Hospital, Guangdong Academy of Medical Sciences, Guangzhou, China; ^2^Guangdong Provincial People's Hospital, School of Medicine, South China University of Technology, Guangzhou, China; ^3^The Second School of Clinical Medicine, Southern Medical University, Guangzhou, China; ^4^Department of Cardiology, Second Clinical Medical College of Jinan University, Shenzhen People's Hospital, First Affiliated Hospital of South University of Science and Technology, Shenzhen, China; ^5^Fujian Provincial Key Laboratory of Cardiovascular Disease, Department of Cardiology, Fujian Provincial Center for Geriatrics, Clinical College of Fujian Provincial Hospital, Fujian Provincial Hospital, Fujian Cardiovascular Institute, Fujian Medical University, Fuzhou, China; ^6^Department of Cardiology, People's Hospital of Baoan Shenzhen, Shenzhen, China; ^7^Department of Cardiology, The Second People's Hospital of Nanhai District, Guangdong General Hospital's Nanhai Hospital, Foshan, China; ^8^Department of Biostatistics, School of Public Health, Southern Medical University, Guangzhou, China

**Keywords:** infection, risk score, ST-elevation myocardial infarction, percutaneous coronary intervention, observational study

## Abstract

**Background:** Post-acute myocardial infarction (post-AMI) infection is an infrequent but important and serious complication in patients with ST-segment elevation myocardial infarction (STEMI) treated with percutaneous coronary intervention (PCI). Predicting its occurrence is essential for future prevention. However, little is known about the prediction of post-AMI infection in such patients to date. This study aims to develop and validate a new risk score based on risk factors for early prediction of infection in STEMI patients undergoing PCI.

**Methods:** This prospective, multi-center and observational study assesses the predictive value of risk score for post-AMI infection among a cohort of patients hospitalized due to STEMI. The STEMI patients undergoing PCI enrolled between January 1st 2010 and May 31st 2016 were served as a development cohort while those enrolled from June 1st 2016 to May 31st 2018 were served as validation cohort. The primary endpoint was post-AMI infection during hospitalization, defined as infection requiring antibiotics (reflecting the clinical influence of infection compatible with the necessity for additional treatment), and all-cause death and major adverse cardiovascular events (MACE) including all-cause death, recurrent myocardial infarction, target vessel revascularization, and stroke were considered as secondary endpoints. The risk score model based on risk factors was established using stepwise logistic regression, and will be validated in other centers and external patients with non-ST-elevation acute coronary syndrome (NSTE-ACS).

**Results:** This study will provide evidence on prognostic property, reliability of scoring, comparative performance, and suitability of the novel model for screening purpose in order to be recommended for clinical practice.

**Discussion:** Our study is designed to develop and validate a clinical risk score for predicting infection in participants with STEMI who have undergone PCI. This simple tool may therefore improve evaluation of post-AMI infection and enhance future researches into the best practices to prevent or reduce infection in such patients.

**Clinical Trial Registration:** www.chictr.org.cn, identifier: ChiCTR1900028278.

## Introduction

ST-segment elevation myocardial infarction (STEMI) accounts for one-third of patients with acute coronary syndrome (ACS) worldwide, which resulted in poor outcomes and enormous financial losses every year (1). Post-acute myocardial infarction infection (post-AMI) is uncommon but is an important and serious complication in such patients. In addition, infections are crucial causes of morbidity and mortality and are associated with prolonged length of hospital stay and a substantial increase in health care costs (2, 3). Taking these undesirable effects into consideration, infection prevention has been one of the highest priorities for medical resources and government. Given that most infections are preventable, identification of STEMI patients at high-risk of infection could allow interventions to prevent and rapidly treat these infections among these patients.

Although some risk-prevention tools for mortality have been developed for STEMI patients, such as the Global Registry of Acute Coronary Events (GRACE) score (4) and the Thrombolysis in Myocardial Infarction (TIMI) risk score (5), there is no specific risk score for identifying STEMI patients undergoing percutaneous coronary intervention (PCI) to estimate the risk of post-AMI infection. In recent articles, we validated the age, creatinine and left ventricular ejection fraction (ACEF) risk score (6) and the Canada Acute Coronary Syndrome (CACS) risk score (7) for post-AMI infection, however, these scores were not developed specially for infection and the discriminatory accuracy were relatively low. In addition, various risk systems have been developed to predict infections following cardiac surgery (8, 9). Fowler et al. established a model that can identify patients undergoing cardiac surgery who were at high- risk for major infection (9). However, no study to date has validated a simplified scoring system to assess patients' individual risk of infection in STEMI patients undergoing PCI.

Given these data, this research protocol aims to develop and validate a practical risk score to identify the risk of post-AMI infection in patients with STEMI undergoing PCI. And this new risk score based on seven simple independent predictors would be divided into four levels according to the corresponding marks (low-, moderate-, high-, and very high-risk groups) to improve the clinical utility. Therefore, the present risk score could be easily applicable in clinical practice for identifying patients at high-risk of infection and in-hospital outcomes in patients with STEMI undergoing PCI and may help clinical decision-making allowing for timely measures to improve clinical outcomes.

## Materials and Methods

### Overview

We will follow the recommended procedures by the Prognosis Research Strategy (PROGRESS) Group (10–12) and show results according to the TRIPOD (Transparent Reporting of a Multivariable Prediction Model for Individual Prognosis or Diagnosis) statement (13, 14). In this research, we develop the risk score and perform the internal validation in patients admitted to Guangdong Provincial People's Hospital, and external validation is conducted in other centers and NSTE-ACS patients.

### Study Design

**Figure 1:**
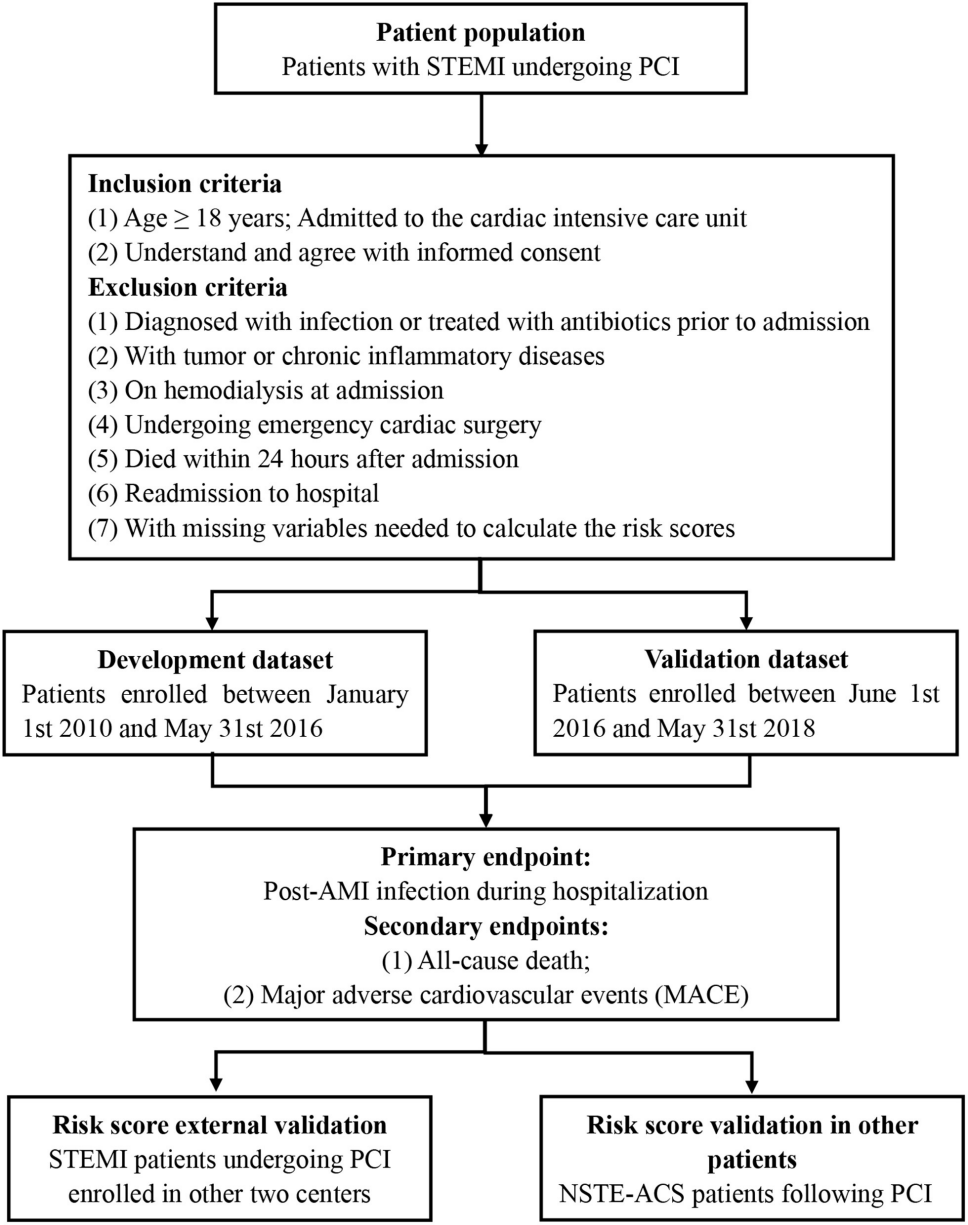
Flow chart of study design.

This proposed protocol involves a prospective, multi-center, observational cohort study (Figure 1). This study expected for enrollment from January 1st 2010 to May 31st 2018. Our study protocol has been approved by the ethics and research committee of Guangdong Provincial People's Hospital (GDREC2016378H) and was registered in the Chinese Clinical Trial Registry (www.chictr.org.cn, ChiCTR1900028278). This study will be designed and performed in accordance with the Code of Ethics of the 1964 Declaration of Helsinki and its later amendments. All enrolled patients will be required to provide written informed consent in this study. Contact information will be provided for participants for further enquiry.

### Study Oversight

The data safety committee has been guaranteed for this research, consisting of several cardiologists and one statistician. The cardiologists will be responsible for the review of the in-hospital course of the participants, while the statistician will conduct the safety analysis, and the data will be divided into development and validation cohorts.

### Study Population

STEMI was defined as the manifestation of typical chest pain and concomitant symptoms for ≥ 30 min but <12 h, with ST-segment elevation ≥ 1 mm in ≥ 2 continuous leads or new or undetermined duration of left branch bundle block accompanying with ≥ 2 times increase in troponin I or T (15).

The inclusion criteria were as follows: (a) age ≥ 18 years; patients with STEMI undergoing PCI, admitted to the cardiac intensive care unit; (b) patients could understand and agree with informed consent.

The exclusion criteria were as follows: (a) patients diagnosed with infection or treated with antibiotics prior to admission; (b) patients with tumor or chronic inflammatory diseases; (c) patients on hemodialysis at admission; (d) patients undergoing emergency cardiac surgery; (e) patients died within 24 h after admission; (f) readmission to hospital; and (g) patients with missing variables that are needed to calculate the risk scores.

Eligible patients were consecutively enrolled between January 1st 2010 and May 31st 2016, which were served as a development cohort. In accordance with the inclusion and exclusion criteria, patients enrolled from June 1st 2016 to May 31st 2018, with complete data regarding risk factors identified in the development cohort, will be served as a validation cohort. Furthermore, the patients screened according to the criteria in other centers will be enrolled for external validation.

To extend the application, this risk score will also be validated in our previous study including patients diagnosed with non-ST-elevation acute coronary syndrome (NSTE-ACS) following PCI according to the same exclusion criteria (16). This retrospective cohort study was designed to evaluate the association between parenteral anticoagulation therapy and clinical outcomes in patients mentioned above. Patients with principal discharge diagnosis of non-ST elevation myocardial infarction (NSTEMI) or unstable angina (UA) and underwent PCI during the index hospital stay were included. And patients with elevated cardiac biomarker and without new ST-elevation on ECG prior to index PCI were classified as NSTEMI while those were classified as UA if there were no positive cardiac biomarker findings and without new ST-elevation on ECG prior to index PCI (16).

### Laboratory Testing

Blood samples were obtained routinely and tested for electrolytes, white blood cell count, hemoglobin, cardiac enzymes, serum creatinine, blood lipids, serum albumin, total bilirubin, estimated glomerular filtration rate (eGFR) that was evaluated using the modified Modification of Diet in Renal Disease (MDRD) equation for Chinese people (17) and other routine laboratory parameters with 24 h after admission, while the medical history, the vital signs including body temperature, pulse rate, respiration rate and blood pressure, and Killip classification were also recorded at the same time.

### Procedure and Medications

All eligible participants received a conventional chest X-ray and ultrasonic cardiography. The left ventricular ejection fraction (LVEF) was measured by echocardiography after admission. Furthermore, blood cultures were examined at the discretion of the responsible physicians. Before the procedure, administration of 300 mg aspirin and 180 mg ticagrelor or clopidogrel (300 or 600 mg) were mandatory for all included patients. PCI was performed by the interventional cardiologists in the conventional manner, and coronary stents were, or other procedures were used when required. Hydration with intravenous normal saline solution at a hydration rate of 1 ml/kg/h or 0.5 ml/kg/h if the LVEF was <40% was initiated during the procedure and maintained until 6 to 12 h after the procedure. After completion of this procedure, lifelong aspirin (100 mg/day) was prescribed and at least 12 months of clopidogrel (100 mg/day) or ticagrelor (90 mg twice/day) were recommended to all patients. Additionally, the prescription of anticoagulants, β blockers, glycoprotein IIb/IIIa inhibitors, or angiotensin-converting enzyme inhibitors/ angiotensin receptor blockers (ACEI/ARB) was at the discretion of the operators according to current guidelines.

### Data Collection and Missing Data

Clinical data including patient demographics, diagnosis, medical histories, clinical and laboratory characteristics, medication treatment, procedural details and main outcomes were obtained through review of electronic medical records. The principal data, such as clinical outcomes, were collected by two investigators independently, and inconsistent data were verified by a third investigator.

Variables were included as candidates for this risk score after removing variables with ≥ 10% missing values (Table 1), and for those with incomplete data, we assumed that patients with the missing data ≤ 5% occurred at random depending on the clinical variables and used multiple imputations. This risk score model was developed based on imputed complete cases.

**Table 1:** Observations and endpoints.

**Category**	**Method and materials**	**Observation and examination items**
Demographic characteristics	Interview	Age, sex and smoking status.
STEMI	Medical examination and interview	Onset of STEMI, date of hospitalization, description of treatment.
History of present illness/previous history/therapies	Interview	Previous history of cardiovascular and cardiovascular risk-related diseases and history of treatment (Hypertension, MI, CABG, COPD, AF, stroke, PCI, sudden cardiac arrest, thrombolysis, pericardial effusion, PCI lesion).
Physical findings	Medical examination	SBP, DBP, heart rate and Killip class.
Family history	Interview	Coronary artery diseases, hypercholesterolaemia and ischaemic cerebral infarction in relatives to the second degree of kinship.
Hematological and biochemical examinations	Serum and urine	The following parameters will be measured from blood acquired within 24 h after admission: WBC, eGFR, creatinine, albumin, hemoglobin, TG, TC, total bilirubin, direct bilirubin, LDL, HDL, ALT, CO_2_ combining power, Urine PH.
Heart function	Echocardiography	LVEF, LVDd, LVDs.
Medication during hospital stay	Electronic medical record	Statins, Warfarin, Tirofiban, ARB, CCB, ACEI, β-blockers, insulin, metformin, PPI, diuretics and nitrates.
Procedural characteristics	Electronic medical record	PCI approach, multi-lesion, contrast volume, number of stents and total length of stents.
Primary endpoint	Interview	Post-acute myocardial infarction infection during hospital admission and the type of infections (pulmonary infections, urinary infections or others including abdominal sepsis, primary bacteremia, and unidentified primary infection sites).
Secondary endpoints	Interview	Presence or absence events: in-hospital all-cause mortality and major adverse cardiovascular events (MACE) including all-cause death, recurrent myocardial infarction, target vessel revascularization, and stroke.

*STEMI, ST-segment elevation myocardial infarction; MI, myocardial infarction; CABG, coronary artery bypass graft; COPD, chronic obstructive pulmonary disease; AF, atrial fibrillation; PCI, percutaneous coronary intervention; SBP, systolic blood pressure; DBP, diastolic blood pressure; WBC, white blood cell; eGFR, estimated glomerular filtration rate; TG, triglycerides; TC, total cholesterol; LDL, low-density lipoprotein; HDL, high-density lipoprotein; ALT, alanine aminotransferase; LVEF, left ventricular ejection fraction; LVDd, left ventricular end-diastolic diameter; LVDs, left ventricular end-systolic diameter; ARB, angiotensin receptor blockers; CCB, calcium channel blockers; ACEI, angiotensin converting enzyme inhibitors; PPI, proton pump inhibitor*.

### Study Endpoints

#### Primary Endpoint

The primary outcome was post-AMI infection during hospital admission, which was defined as infection requiring antibiotics (reflecting the clinical influence of infection compatible with the necessity for additional treatment) (18). The types of infections included pulmonary infections, urinary infections or others (including abdominal sepsis, primary bacteremia, and unidentified primary infection site) based on the clinical records during hospitalization. Appropriate antibiotics were used once infection was confirmed (19). The antibiotics were validated by the hospital infection control committee that approved the initiation of therapy for each infection case.

#### Secondary Endpoints

The secondary endpoints were in-hospital all-cause mortality and major adverse cardiovascular events (MACE) including all-cause death, recurrent myocardial infarction, target vessel revascularization, and stroke (20) (Table 2). All these adverse events will be recorded in detail during in-hospital period.

**Table 2:** Outcome definitions in MACE.

**Term**	**Definitions**
All-cause death	Any death recorded between the date of enrollment and the end of data linkage.
Recurrent myocardial infarction	Characteristics of MI occur after 28 days following an incident MI^15^.
Target vessel revascularization	Any revascularization procedure involving PCI of the target lesion or surgical bypass of the target vessel.
Stroke	The presence of a new focal neurological deficit thought to be vascular in origin, with signs or symptoms lasting > 24 h.

*MACE, major adverse cardiovascular events; MI, myocardial infarction; PCI, percutaneous coronary intervention*.

### Statistical Analysis

#### Sample Size Consideration

A rule of thumb, namely, that the event per variable (EPV) was 10 or greater under this circumstance was applied for the sample size determination. Given no more than 8 risk factors in developing a predictive model, thus at least 80 infections were needed. According to the previous studies (3), the incidence of infection was 10%, and at least 800 patients should be finally included.

#### Data Analysis

The student's *t*-test or Wilcoxon rank-sum test was used to compare the continuous variables and the chi-square test or Fisher exact test was used as appropriate for categorical variables for the comparison of patients with or without infections in both datasets.

The risk score was developed using a backward stepwise logistic regression model. All candidate predictors were included in the development of the risk score, and to avoid overfitting data and select the best subset of risk factors, a bootstrap method was used. For missing data, 10 multiple imputations were performed using the SAS MI procedure, and 100 bootstrap repeats were conducted for each imputation. Variables selected in at least 90% of 1,000 bootstrap repeats were included in the final model. The risk score was developed for each imputed data via using the selected variables and the SAS MIANALYZE procedure was used to combine the results of the analyses. For this risk score, the scoring method was analogous to that of Sullivan et al. (21) and was used based on the developed risk score. For the scoring purpose, continuous variables were categorized into groups regarding the clinical significance. The risk score of post-AMI consisted of four levels (low-risk, moderate-risk, high-risk, and very high-risk) in order to enhance the clinical utility.

Both discriminatory accuracies assessed by the C-statistic and calibration measured by the Hosmer-Lemeshow χ^2^ statistic and calibration plot were used to evaluate the predictive accuracy of the risk score. The bootstrap method with 1,000 bootstrapped replications was used to perform an internal cross-validation of the risk score. The average of the C-statistic was reported. In addition, external validation and subgroup validation (older, gender, diabetes, anemia, and chronic kidney disease) of the risk score model was conducted to assess the stability of the model. The risk score was also validated in patients with NSTE-ACS undergoing PCI, and the area under curve (AUC) of this risk score was compared to the previous scoring systems (6, 7). Furthermore, comparison of the area under the receiver operating characteristic (ROC) curve was according to the nonparametric approach of DeLong et al. (22). To evaluate the clinical utility of this risk score, decision curve analysis was introduced and the risk score with a higher net benefit indicated a better clinical effect (23).

All statistical analyses were performed using SAS (version 9.4, SAS Institute, 210 Cary, North Carolina, USA).

## Discussion

The results of this study will develop and validate a relatively simple risk score based on easily acquired clinical variables to identify the patients with STEMI undergoing PCI at high-risk of post-AMI infection. In addition, the predictive accuracy of this risk score will be validated in several subgroups and an external validation cohort including patients with NSTE-ACS.

Infection is known to have unacceptably high and alterable mortality. Thus, early risk stratification of patients is a crucial clinical task. Some studies have reported the risk scores or markers to assess the probability of infection in patients with cardiovascular diseases or in critically ill patients. Fowler et al. developed a simplified risk score with twelve variables to identify high-risk for major infection in patients undergoing cardiac surgery (9). The variables included in this risk score were age, body mass index (BMI), diabetes, renal failure, congestive heart failure, peripheral vascular disease, female gender, chronic lung disease, cardiogenic shock, myocardial infarction, concomitant surgery and intraoperative variables including perfusion time and intra-aortic balloon pump. The model that limited to preoperative characteristics and combined model including both preoperative and intraoperative characteristics achieved a *C*-index of 0.697 and 0.708, respectively. Therefore, the discriminatory accuracies of these prediction models were relatively low, and the model only reflected the overall clinical objective due to broader definitions (9). Raja SG et al. reported that the Brompton Harefield Infection Score (BHIS) could effectively predict surgical site infection (SSI) risks and contribute to risk stratification in patients following coronary artery bypass graft (CABG) (24). The baseline risk score consisting of female, diabetes or HbA1c >7.5%, BMI ≥ 35, LVEF <45% and emergency surgery offered an effective predictive ability (ROC curve was 0.727). However, this model had several limitations, the relatively small number of SSI events (n=96) limited the ability to identify associations with a large number of variables. Additionally, the accuracy and utility of the BHIS score tool were only validated internally but not in an external dataset (24). Furthermore, several other risk score models, such as the intensive care infection score and the clinical pulmonary infection score were established to assess the infection risks in critically ill patients (25, 26). However, their practical utility was limited because of the lower predictive value (25) and the circumscribed application to pulmonary infection (26). Up to now, few studies have proposed an infection prediction model for patients with STEMI before our recent researches, and there is no scoring system established specifically to assess patients' individual risk of infection among STEMI following PCI. Therefore, this study aimed to develop and validate a novel risk score based on risk factors for early prediction of infection in these patients.

This observational study protocol has several limitations. First, this risk score was exclusive to STEMI patients and validated in patients with NSTE-ACS, clinicians should take special cautions when applying these results to other patients, such as stable coronary artery disease. Second, infection was difficult to diagnose to some extent and would be overestimated sometimes. However, the infection in this study was those treated with antibiotics which were strictly confirmed and approved by the hospital infection control committee. Third, the candidate risk variables are limited to those available in the patient cohort of this study. The more variables the scoring system contains, the higher the predictive accuracy of the risk score achieved. Finally, the current risk score did not include any new biomarkers. Incorporating new biomarkers may create a more robust model. However, this risk score was derived from clinical data routinely collected and would be more accessible in clinical practice.

In conclusion, to the extent of our knowledge, our risk score will be the first risk model for early prediction of infection in patients with STEMI undergoing PCI. This simple tool may therefore improve evaluation of post-AMI infection and enhance future researches into the best practices to prevent or reduce the infection in such patients.

## Ethics Statement

The studies involving human participants were reviewed and approved by Ethics Committee of Guangdong Provincial People's Hospital. The patients/participants provided their written informed consent to participate in this study.

## Author Contributions

PH, NT, and JC: project administration and resources. PH, CD, NT, and YLiu: study design. YLiu and LW: writing-original draft. YLiu, LW, PC, and LX: writing-review and editing. LW, YLin, WC, ZX, PC, YD, LZ, and HF: data collection. All authors contributed to the article and approved the submitted version.

## Conflict of Interest

The authors declare that the research was conducted in the absence of any commercial or financial relationships that could be construed as a potential conflict of interest.
